# GABA_A_ receptor activity shapes the formation of inhibitory synapses between developing medium spiny neurons

**DOI:** 10.3389/fncel.2015.00290

**Published:** 2015-08-06

**Authors:** Jessica Arama, Karine Abitbol, Darren Goffin, Celine Fuchs, Talvinder S. Sihra, Alex M. Thomson, Jasmina N. Jovanovic

**Affiliations:** ^1^UCL School of Pharmacy, University College LondonLondon, UK; ^2^Neuroscience, Physiology and Pharmacology, UCL Division of Biosciences, University College LondonLondon, UK

**Keywords:** GABA_A_ receptors, GABAergic synapses, striatal medium spiny neuron, hyperpolarizing shift, GABA_A_ receptor depolarization

## Abstract

Basal ganglia play an essential role in motor coordination and cognitive functions. The GABAergic medium spiny neurons (MSNs) account for ~95% of all the neurons in this brain region. Central to the normal functioning of MSNs is integration of synaptic activity arriving from the glutamatergic corticostriatal and thalamostriatal afferents, with synaptic inhibition mediated by local interneurons and MSN axon collaterals. In this study we have investigated how the specific types of GABAergic synapses between the MSNs develop over time, and how the activity of GABA_A_ receptors (GABA_A_Rs) influences this development. Isolated embryonic (E17) MSNs form a homogenous population *in vitro* and display spontaneous synaptic activity and functional properties similar to their *in vivo* counterparts. In dual whole-cell recordings of synaptically connected pairs of MSNs, action potential (AP)-activated synaptic events were detected between 7 and 14 days *in vitro* (DIV), which coincided with the shift in GABA_A_R operation from depolarization to hyperpolarization, as detected indirectly by intracellular calcium imaging. In parallel, the predominant subtypes of inhibitory synapses, which innervate dendrites of MSNs and contain GABA_A_R α1 or α2 subunits, underwent distinct changes in the size of postsynaptic clusters, with α1 becoming smaller and α2 larger over time, while both the percentage and the size of mixed α1/α2-postsynaptic clusters were increased. When activity of GABA_A_Rs was under chronic blockade between 4–7 DIV, the structural properties of these synapses remained unchanged. In contrast, chronic inhibition of GABA_A_Rs between 7–14 DIV led to reduction in size of α1- and α1/α2-postsynaptic clusters and a concomitant increase in number and size of α2-postsynaptic clusters. Thus, the main subtypes of GABAergic synapses formed by MSNs are regulated by GABA_A_R activity, but in opposite directions, and thus appear to be driven by different molecular mechanisms.

## Introduction

The basal ganglia control a variety of functions in the brain, including motor coordination and emotional and cognitive information processing (Nelson and Kreitzer, 2014). Functional and structural deficits in this brain region thus have a major impact on health and survival, leading to various neurological and neurodegenerative disorders, including Huntington’s or Parkinson’s disease (Obeso et al., 2014). The central part of the basal ganglia is represented by the striatum, a region composed primarily of GABAergic medium spiny neurons (MSNs, ~95%) and a small number of GABAergic and cholinergic interneurons (~5% of all neurons; Tepper and Bolam, 2004). The activity of MSNs in this region is driven by excitatory glutamatergic inputs received from the cortex and thalamus, and modulated by dopaminergic inputs from the *substantia nigra pars compacta*. This activity is counterbalanced by the local network of GABAergic interneurons forming inhibitory synapses with the MSNs (Tepper and Bolam, 2004; Mallet et al., 2005), and, to a limited extent, by the MSN axon collaterals forming synapses with the neighboring MSNs (Gerfen, 1988; Guzmán et al., 2003; Taverna et al., 2008; Chuhma et al., 2011). However, the vast majority of MSN axons form inhibitory synapses with targets outside of the striatum (Tepper et al., 2007). The finely tuned network of inhibitory connections within the striatum plays a key role in setting up the inhibitory tone of MSN projections, thereby determining the final functional output of the whole basal ganglia region. This raises a question as to how the synapses between MSNs in the striatum are formed during development and what regulates the extent of their connectivity. Although the origin and migration of MSNs from the lateral ganglionic eminence, starting around the embryonic day 12 in rodents (Fishell and van der Kooy, 1991; Deacon et al., 1994; Olsson et al., 1995; Anderson et al., 1997; Toresson and Campbell, 2001; Yun et al., 2003), have been extensively studied, little is currently known about the regulation of MSN development within the primordial striatum and formation of synaptic connections with their targets.

The extent of collateral inhibition between the embryonic MSNs in primary culture is negatively regulated by dopamine through the activation of D1 and D2 receptors. This regulatory mechanism involves a decrease in the size of synaptic GABA_A_ receptor clusters and a reduction in the number of GABAergic synapses formed between the MSNs (Goffin et al., 2010). Dopamine and dopamine receptors are abundant in the embryonic striatum and the first dopaminergic projections from the *substantia nigra* are formed as early as E12.5 (Voorn et al., 1988; Gates et al., 2006), which correlates tightly with the timing of MSN migration from the proliferative zones to this region. If dopamine plays a prominent role in MSN development, the question remains whether other classical neurotransmitters also take part in these regulatory processes. During embryonic development, glutamate and acetylcholine are not very abundant in the striatum because glutamatergic inputs to the striatum (Dehorter et al., 2011; Sohur et al., 2014), as well as cholinergic interneurons (Aznavour et al., 2003) mostly develop after birth during the first postnatal week (P7) or later. However, together with dopamine, as a developmental signal exogenous to the striatum, GABA as the principal endogenous neurotransmitter may also be involved in these regulatory processes.

The role of GABA as a developmental signal has been well established in many other brain regions, where activation of GABA_A_ receptors regulates multiple developmental processes, including neurite extension and synaptogenesis (Akerman and Cline, 2007; Dehorter et al., 2011; Ben-Ari et al., 2012; Deidda et al., 2014). GABA_A_ receptors are members of a diverse family of hetero-pentameric GABA-gated chloride/bicarbonate channels, which can be assembled from several classes of homologous subunits: α (1–6), β (1–3), γ (1–3), δ, ε, θ and π (Sieghart, 2006). The structural diversity of GABA_A_ receptors has long been recognized as being a key determinant of the wide range of their functional and pharmacological properties (Mohler et al., 1995; Whiting, 2003). Although all synaptic GABA_A_ receptors typically contain two α subunits (α1, 2, 3 or 5), two β subunits (β2 or β3) and a γ2 subunit, the type of the α subunit present determines the affinity for GABA and kinetic properties of these receptors, as well as their subcellular localization, incorporation into specific types of synapses (Klausberger et al., 2002; Thomson and Jovanovic, 2010), and drug sensitivity (Möhler, 2015). While specific synaptic distribution of α subunits in the adult basal ganglia has been reported (Gross et al., 2011), it is currently unknown how these specific synapses are formed during development, whether they include synapses formed by MSN collaterals, and whether the activity of GABA_A_ receptors plays a regulatory role in synapse formation. In the current study, we have examined synaptic development within the population of embryonic MSNs, first, by characterizing changes in the GABAergic synaptic activity of these neurons, and, second, by performing detailed structural analysis of α1- and α2-containing synaptic connections under conditions of chronic GABA_A_ receptor blockade.

## Materials and Methods

### Primary Neuronal Cultures

Sprague-Dawley rats (Harlan, UK; the number of pregnant females used was ~30) were housed and sacrificed according to UK Home Office [and European Communities Council directive of 24 November 1986 (86/609/EEC)] guidelines. The project was formally approved by the UCL School of Pharmacy Ethics Committee.

Primary cultures of MSNs were prepared as described previously (Banker and Goslin, 1988; Goffin et al., 2010). Striata were dissected from embryonic day 16–17 (E16–17) Sprague-Dawley rats (Harlan UK), dissociated by trituration in Ca^2+^ and Mg^2+^-free Hepes-buffered saline solution (HBSS; Invitrogen, USA), plated at a density of 45,000 cells per cm^2^ in neurobasal medium, containing B27 supplement, glutamine (2 mM), penicillin (100 Units), streptomycin (100 μg) and glucose (6 mM; all from Invitrogen) on 0.1 mg/ml poly-L-lysine- and 0.01 mg/ml laminin-coated glass coverslips or glass bottom dishes (Mattek, USA). Cultures were incubated in a humidified 37°C/5% CO_2_ incubator for 7 or 14 days prior to experimentation.

### Immunocytochemistry

Cultured MSNs were fixed with 4% paraformaldehyde/4% sucrose/phosphate buffered saline (PBS) for 15 min. Cultures were washed and incubated with 1% bovine serum albumin (BSA)/PBS for 30 min to reduce non-specific binding. For characterization of neuronal cell types present in the culture, immunocytochemistry was done using the following primary antibodies: anti-GABA (rabbit polyclonal, 1:000 dilution; Sigma, USA), anti-Glial Fibrillary Acidic Protein (GFAP; mouse monoclonal, 1:600 dilution, Sigma, USA), anti-MAP2 (rabbit polyclonal, 1:1000 dilution, Sigma), anti-Dopamine-and cAMP-regulated phosphoprotein, Mr 32 kDa (DARPP-32; mouse monoclonal, 1:2000 dilution, Hemmings and Greengard, 1986), anti-Parvalbumine (rabbit polyclonal, 1:2000; Swant, Switzerland) and anti-Neuropeptide Y (NPY; rabbit polyclonal, 1:2000, Immunostar, USA). For analysis of GABAergic synapses, cultures were first incubated with anti-GABA_A_ receptor α_1_ (rabbit polyclonal, 1:500, (Duggan and Stephenson, 1990), α_2_ (guinea pig, 1:1000, Synaptic Systems, Germany) or β_2/3_ subunit (MAB341, bd17 clone, 10 μg/ml, Merck Millipore, USA)-specific primary antibodies in 1% BSA/PBS overnight (14–16 h) at 4°C, without permeabilization. Cultures were then washed with PBS and cells permeabilized with 0.2% Triton X-100/PBS for 30 min, followed by incubation with 1% BSA/PBS for 30 min, to block non-specific binding. Cultures were then incubated with mouse anti-glutamate decarboxylase (GAD)-65 antibody (1:3000; Merck Millipore, UK), or rabbit anti-vesicular inhibitory amino acid transporter (VIAAT) antibody (1:1000, Dumoulin et al., 2000), or mouse anti-gephyrin antibody (1:1000, Synaptic Systems, Germany; clone 311B (Hoon et al., 2011; Nair et al., 2013) and chicken anti-MAP2 antibody (1:1000, Abcam) for 60 min. Primary antibodies were visualized after staining with the appropriate goat anti-mouse, anti-rabbit or anti-guinea pig Immunoglobulin G (IgG) conjugated to AlexaFluor488, AlexaFluor555, Cy5 or AlexaFluor647, respectively (1:750, Life Technologies), in 1% BSA/PBS for 60 min. Cultures were washed and coverslips mounted using ProLong antifade reagent (Life Technologies). Immuno-reactivity was visualized using laser scanning confocal microscope (Zeiss LSM 710 Meta, Germany) with ×40 or ×63 oil-immersion objective. In all experiments in which synaptic parameters (the number and size of GABA_A_ receptor clusters and the number of presynaptic terminals) were analyzed and compared between different groups, all the neurons were always obtained from the same tissue and platted at the same density. They were all immunolabeled with the same antibodies under the same experimental conditions. Likewise, confocal imaging of neurons in different groups was always done with the same laser power and detector settings, which were adjusted to avoid any saturated levels.

### Quantification of Puncta Area, Number and Co-Localization

For each treatment, a minimum of 16 randomly selected neurons were examined in at least two independent experiments. For these experiments, the number, area and co-localization of puncta were determined from confocal images using the LSM Image Programme. Immunopositive puncta were defined as immuno-reactivity greater than 0.1 μm^2^ present along the first 20 μm length of primary processes (Yu et al., 2007; Goffin et al., 2010). The threshold for detection for each channel in each image was calculated as the mean pixel intensity for the entire image plus two standard deviations above the mean. The threshold did not vary significantly between different images within the experiment (<10%). Puncta co-localization in two different fluorescence channels was determined by overlaying the images. A GABA_A_ receptor positive cluster was considered to co-localize with a GAD-65-positive presynaptic terminal, resulting in a yellow color, when 50% or more of the labeling overlapped. Similarly, gephyrin-positive clusters were considered to be co-localized with GABA_A_ receptor-positive clusters when 50% or more of the labeling overlapped. Finally, mixed α1/α2 GABA_A_ receptor clusters were also defined based on the minimum of 50% overlap in labeling between separate channels. The number, size and co-localization were analyzed using Excel and Origin Pro 9.1 software, and the values were expressed as medians and their interquartile range (IQR). Normality tests were performed using the Shapiro-Wilk and Kolmogorov-Smirnov tests. After normality tests were performed on each of the groups, non parametric statistical analysis was done using Mann-Whitney test with the confidence interval of 95%, as the groups showed non-Gaussian distribution.

### Intracellular Ca^2+^ Imaging Using Fluo-4-AM

MSNs (7 or 14 days *in vitro*, DIV) plated on glass-bottom dishes (Mattek, USA) were loaded with the Ca^2+^ indicator Fluo-4-AM (5 μM, Invitrogen) by bath application for 30 min at 37°C in loading buffer (in mM: 10 HEPES, pH 7.4, 150 NaCl, 3 KCl, 2 MgCl_2_, 0.1 CaCl_2_, 5 Glucose), in the presence of Tetrodotoxin citrate (TTX, 0.5 μM, Ascent Scientific Ltd.), (D-(-)-2-Amino-5-phosphonopentanoic acid (D-AP5, 50 μM, Tocris) and 6, 7-dinitroquinoxaline-2, 3-dione (DNQX, 20 μM, Tocris). Muscimol (50 μM, Tocris) was applied to the bath in the presence or absence of bicuculline methochloride (Bic 50 μM, Ascent Scientific Ltd.) or picrotoxin (Pic; 50 μM, Tocris). TTX, D-AP5, muscimol and bicuculline were prepared as concentrated stocks in water, while DNQX and Pic stocks were prepared in Dimethyl sulfoxide (DMSO) and ethanol, respectively. The final DMSO or ethanol concentrations had no effect on intracellular Ca^2+^ levels. Ca^2+^ responses were recorded at 37°C as digitized images acquired using a Nikon Diaphot 300 inverted microscope with epifluorescence attachments. Data were collected with a Hamamatsu ORCA 2-cooled CCD camera using MetaFluor software (Universal Imaging) with images acquired every 5 s. The series of digitized fluorescence images was analyzed by MetaFluor software to determine the average level of fluorescence of each cell at each time point sampled as described before (Porcher et al., 2011). ΔF was calculated as following: the basal fluorescence (F_bas_) was obtained by averaging the neuronal fluorescence intensity of the last five frames before application of muscimol. Frame fluorescence (F_fr_) was the neuronal fluorescence intensity of the frame. Subsequently,ΔF = (F_fr_ − F_bas_) was expressed as percentage of maximum fluorescence detected following the addition of 50 mM KCl to the bath. The peak value of ΔF following muscimol application in the absence or presence of Bic or Pic was used for the statistical analysis.

### Electrophysiology

MSNs were recorded in whole-cell mode (using IR-DIC optics, Olympus BX51). The extracellular medium contained (in mM) 130 NaCl, 4 KCl, 10 HEPES, 20 NaHCO_3_, 10 glucose, 1 MgCl_2_ and 2 CaCl_2_, equilibrated with 5% CO_2_/95% O_2_ (pH 7.4, 330 mosmol.l^−1^) at 32°C (flow rate 1.8 ml.min^−1^). Whole cell pipettes had a final resistance of 3–8 MΩ when filled with intracellular solution, in mM: 130 KCl, 3 NaCl, 4.5 phosphocreatine, 10 HEPES, 1 EGTA, 3.5 Na-ATP, 0.45 Na-GTP and 2 MgCl_2_, adjusted to pH 7.2 with KOH, 290–300 mosmol.l^−1^. Recordings were discarded if access resistance exceeded 15 MΩ. The selection of cells for the analysis was made based on the quality and duration of recordings obtained. In many recordings the frequencies were too low and recordings too brief to allow confident analysis of enough events. The recordings selected for the analysis were the longer recordings of highest quality. Action potential (AP) amplitudes, widths at half amplitude, and AP afterhyperpolarization (AHP) amplitudes, were measured in current clamp mode, from the first AP triggered by a just suprathreshold depolarizing current pulse. Membrane time constants (τ_m_) were measured from the decay of the response to a subthreshold depolarizing current pulse from a membrane potential of −70 mV. Continuous recordings of spontaneous synaptic events, recorded in voltage clamp mode, were filtered at 5 kHz, digitized at 10 kHz and collected with Spike2 (CED 1401, Cambridge Electronic Design), from a holding potential of −70 mV. Putative inhibitory postsynaptic currents (IPSCs) were detected off-line according to a current threshold and selected (manually, by shape) for further analysis (MSpike, D.C. West). Computed averages of miniature IPSCs (mIPSCs), spontaneous IPSCs (sIPSCs) and AP-IPSCs used the fast rising phase of the IPSC, or the fast rising phase of the presynaptic AP (dual recordings) as trigger. The shape of the average IPSC and timing of the peak, relative to the presynaptic spike, informed individual manual IPSC amplitude measurements. Wherever possible all spontaneous events were measured for the amplitude distribution plots, but the decay phases of some were contaminated by other spontaneous events. For averaged records (Figures 4B,C,F,G) therefore, those events that were uncontaminated for their entire time course were selected. The histograms and bar graphs give the more complete picture of IPSC amplitudes, but the shape and time course are better represented by averages of “clean” events. Standard deviation time course (SDTC) was computed in parallel with each average, to ensure that the events included in averages were of similar shape, i.e., were largely devoid of confounding spontaneous events and artifacts, and that triggers were accurately aligned for all contributory sweeps, before further analysis. Following collection of sIPSCs under control conditions in the presence of D-AP5 (50 μM) and DNQX (20 μM), mIPSCs were recorded in the presence of TTX (1 μM). In some experiments, bicuculline methochloride (10 μM) was then added. IPSC 10–90% rise time (RT) and width at half amplitude (HW) were measured from averages and IPSC amplitude distributions were constructed from single event measurements. Paired whole cell recordings were made from MSNs. APs were elicited with injected depolarizing current pulses in one neuron (current clamp recordings) and postsynaptic responses recorded in the other (voltage clamp recordings, from a membrane potential of -70 mV). For figures, some electrophysiological traces were smoothed (3 point running average) to reduce high frequency noise and enhance clarity. PSI-plot (Poly Software International), GraphPad Prism (GraphPad Software, Inc.), Excel (Microsoft) and OriginPro 9.1 were used for data plotting and statistical analysis. Unless otherwise stated, data are given as means and standard error of the mean (s.e.m.). Differences between means were tested with Student’s *t* test.

## Results

### Characterization of Developing MSNs in Culture

**Figure 1:**
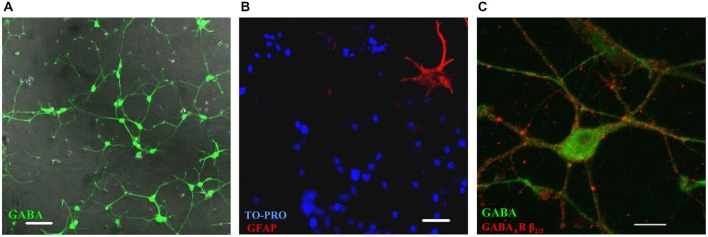
**Embryonic (E17) medium spiny neurons (MSNs) form a homogenous population of GABAergic neurons *in vitro*. (A)** Cultures are almost 100% immunoreactive for the neurotransmitter GABA (green; DIC image was overlaid on top. Scale bar = 50 μm). **(B)** Glial fibrillary acidic protein (GFAP)-positive astrocytes (red) are present in cultures but only in small numbers compared to total number of cells assessed by the nuclear stain TO-PRO (blue). Scale bar = 50 μm. **(C)** Image of a MSN showing the intracellular staining for GABA (green) and the cell surface staining for GABA_A_ receptor β2/3 subunits (red). Scale bar = 10 μm. Representative images from two independent experiments.

MSN precursors isolated from the embryonic (E16–17) basal ganglia regions form a homogenous population of GABAergic neurons in primary culture. The vast majority of these cells were labeled with a GABA-specific antibody (Figure 1A), while only 3.2 ± 0.7% of cells were immunoreactive for GFAP, a marker of glia (Eng et al., 2000; Figure 1B). The latter figure is likely to be an over-estimation as quantification was performed only in rare areas that contained at least one GFAP-positive cell. These GABAergic neurons develop elaborate dendritic processes during the first week in culture, and express and cluster GABA_A_ receptors at the cell surface as revealed by immunolabeling with a GABA_A_ receptor β_2/3_ subunit-specific antibody (Figure 1C).

**Figure 2:**
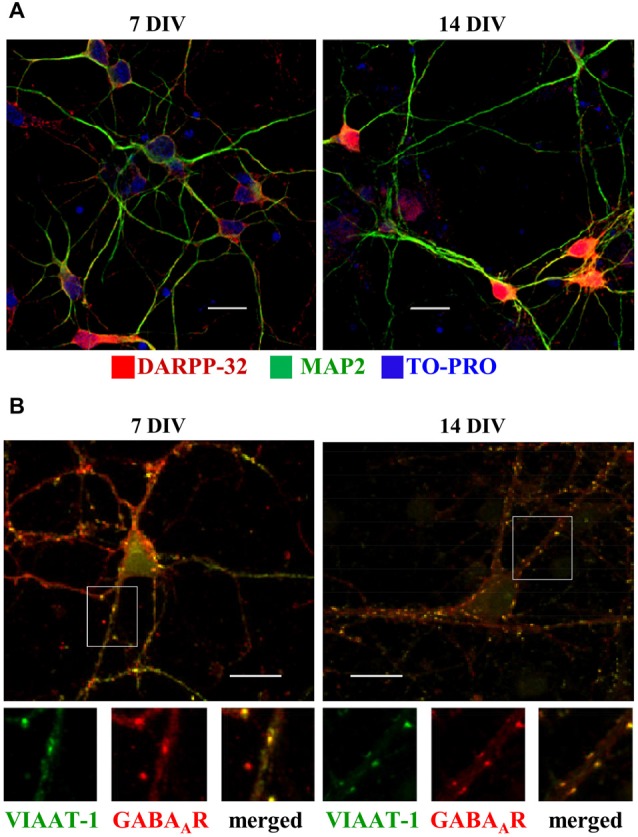
**Developmental changes in embryonic MSNs from 7 to 14 days *in vitro* (DIV). (A)** Expression of Dopamine- and cAMP-regulated phosphoprotein, Mr 32 kDa (DARPP-32, red), detected in MSNs labeled with MAP2-specific antibody (green) and nuclear stain TO-PRO, is prominently increased from 7 (*left*) to 14 (*right*) DIV. Scale bar = 20 μm. **(B)** Increase in the number of synaptic contacts (yellow, *small right panel*) received by MSNs cultured from 7 (*left*) to 14 (*right*) DIV, as detected by colocalization of immunolabeled postsynaptic GABA_A_ receptor β2/3 subunit clusters (red, *small middle panel*), and presynaptic vesicular inhibitory amino acid transporter (VIAAT)-1 terminals (green, *small left panel*). Representative images from three independent experiments. Scale bar = 20 μm (*upper panels*).

At 7 DIV, the vast majority of the neurons in culture were immunopositive for DARPP-32, a well-established marker of MSNs (Svenningsson et al., 2004) and MAP2 (Figure 2A). A small population of NPY/MAP2-positive neurons was detected at 7 DIV (3.1 ± 1.2% of neurons) and 14 DIV (4.6 ± 1.1% of neurons; data not shown), while there was no indication that any of the neurones present in the cultures were positive for parvalbumin. As mentioned above, the later figures are likely to be an over-estimation as quantification was performed only in rare areas that contained at least one NPY-positive neuron. In addition, rare DARPP-32 positive cells were identified which were not MAP2-positive, suggesting that they represent glia cells, as demonstrated previously (Hökfelt et al., 1988). Furthermore, DARPP-32 positive neurites, which were not MAP2-positive, likely represent the axons of medium spiny neurons (Gustafson et al., 1989).

The expression of DARPP-32 increased significantly between 7 and 14 DIV, suggesting rapid development and maturation. Increase in DARPP-32 expression coincided with an increase in synaptic connectivity between developing MSNs, which was first revealed by double labeling with GABA_A_ receptor β_2/3_ subunit- and VIAAT-specific antibodies. Confocal imaging demonstrated close appositions between postsynaptic GABA_A_ receptor clusters and VIAAT-containing presynaptic terminals in many locations along dendrites and in somatic regions of MSNs, a hallmark of putative synaptic contacts (Figure 2B).

### Functional Maturation of MSNs

**Figure 3:**
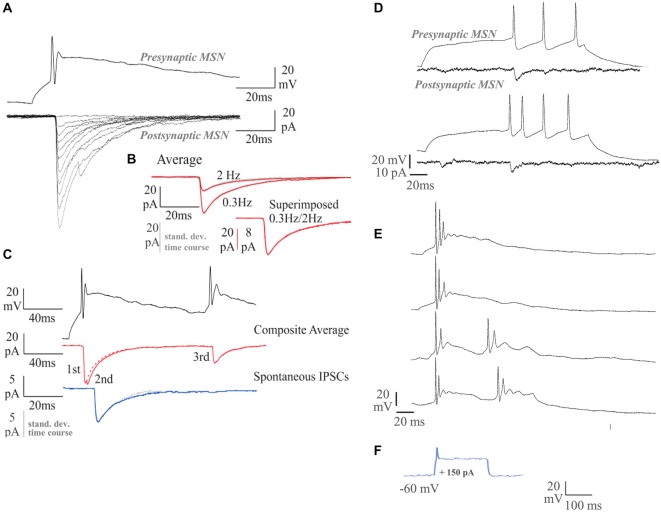
**Dual whole-cell recordings and firing characteristics of synaptically connected MSNs**. The MSNs recordings were carried out at 14 DIV. **(A)** Single sweep inhibitory postsynaptic currents (IPSCs; *lower traces*; Vm = −70 mV) elicited by single spikes in the presynaptic MSN (*upper trace*). **(B)** Average IPSCs elicited by single spikes at 0.3 Hz, or 2 Hz (red), with standard deviation time course (SDTC, gray). The 0.3 Hz and 2 Hz average scaled and superimposed (*lower traces*; scale bar 8 pA for the 0.3 Hz). The similar time course of average and SDTC, and of averages obtained at different firing rates, indicates that all events included had a similar shape. **(C)** Average IPSCs elicited by two spike pairs. Average spontaneous IPSCs (sIPSCS, blue) with SDTC (gray) scaled and superimposed (5 pA scale bar; *lower traces*). **(D)** A near tonically firing MSN responding to sequential depolarizing current pulses, recorded at 14 DIV. Single sweeps of the responses of the simultaneously recorded postsynaptic MSN are shown below. **(E)** A MSN recorded at 16 DIV, displays more mature burst-firing behavior than the cell recorded at 14 DIV. Longer depolarizing current pulses elicit repetitive bursts (lower two records). **(F)** Response to 150 pA current injection in 7 DIV MSN.

Between 5–7 and 12–14 DIV, the proportion of medium spiny neurons that supported recognizable APs increased from <50 to >90%, and by 14 DIV, approximately half the neurons recorded could fire repetitively in response to a suprathreshold, 200 ms depolarizing current pulse. In some of these neurons, near tonic firing of APs was elicited. APs declined by 10 and 20% in amplitude between the first and second AP, at interspike intervals of 30–50 ms, but repetitive single spike firing was maintained throughout the pulse. In other neurons, stereotypical “bursts” of two to three APs with an underlying depolarizing envelope were elicited by just suprathreshold pulses. Repetitive bursts were elicited at interburst intervals of around 100 ms (Figures 3A,C–E). At the very short interspike intervals within these bursts, AP amplitude declined dramatically, to 20–30% of first spike amplitude. That the second depolarizing event in the “burst” is indeed a significantly attenuated AP and not simply an ADP, is demonstrated by its ability to activate a second IPSC (Figure 3C).

Between 5–7 and 12–14 DIV, APs increased in amplitude (15.8 ± 8.4 mV at 5–7 DIV to 43.7 ± 14.4 mV at 12–14 DIV, mean ± SD; *p* < 0.001; *n* = 10) and decreased in duration (width at half amplitude: 3.9 ± 0.9 ms at 5–7 DIV to 1.9 ± 1.1 ms at 12–14 DIV; mean ± SD; *p* < 0.001; Figure 3F). In older cultures, APs in tonically firing neurons, elicited by just suprathreshold pulses, arose from a slow depolarizing ramp typical of MSNs (Nisenbaum et al., 1994). AP AHP increased in amplitude (2.6 ± 3.8 mV at 5–7 DIV to 13.2 ± 5.5 mV at 12–14 DIV, mean ± SD; *p* < 0.001) and membrane time constants increased in duration (3.8 ± 2.5 ms at 5–7 DIV to 6.6 ± 4.1 ms at 12–14 DIV, mean ± SD; *p* = 0.05; data not shown). Correlations (*r* > 0.63) were found between DIV and AP amplitude, DIV and AP width at half amplitude and between AP amplitude and AP width at half amplitude. Correlations (0.35 > *r* > 0.32) between DIV and AHP amplitude and between DIV and τ_m_ were less strong, but indicated that during the second week *in vitro*, APs became steadily taller and narrower, despite an increase in membrane time constants, and elicited increasingly large spike-AHPs or AP-AHPs. It should be noted that a range of properties was apparent at all time points investigated, with more and less apparently mature neurons in a given coverslip.

#### Action Potential-Driven IPSCs in Paired Recordings

To test the ability of the synaptic connections made in these cultures to support AP-driven transmitter release, paired recordings were performed. None of the 32 pairs recorded in younger, 5–7 DIV cultures demonstrated postsynaptic responses that could be elicited by APs in the simultaneously recorded neuron. This suggests that, at this early stage, despite their ability to support spontaneous, single vesicle release, few terminals are sufficiently mature to support AP-driven transmitter release. Of 20 older, 12–14 DIV MSN pairs tested, however, four clearly supported AP-driven release. AP-IPSC amplitudes fluctuated strongly from sweep to sweep (see Figure 3A) and coefficients of variation were relatively large (mean ± SD: 0.6 ± 0.3, range 0.3–1.0). AP-IPSC average amplitudes (mean ± SD: −75.3 ± 41.2 pA, range −41.2 to −96.6 pA) were similar to those of the larger sIPSCs recorded from 12–14 DIV cultures (Figure 3C). Their time course (RT 1.2 ± 0.8 ms; HW 12.2 ± 2.9 ms; mean ± SD) was also similar. In a fifth pair, APs in one medium spiny neuron resulted in a small amplitude event of approximately 20 pA, at constant, short latency, but its time course was obscured by larger, longer latency events. That these later events were consistent in onset latency and time course was indicated by the shape of the SDTC matching that of the average IPSC (Figures 3B,C).

#### Spontaneous Synaptic Activity

**Figure 4:**
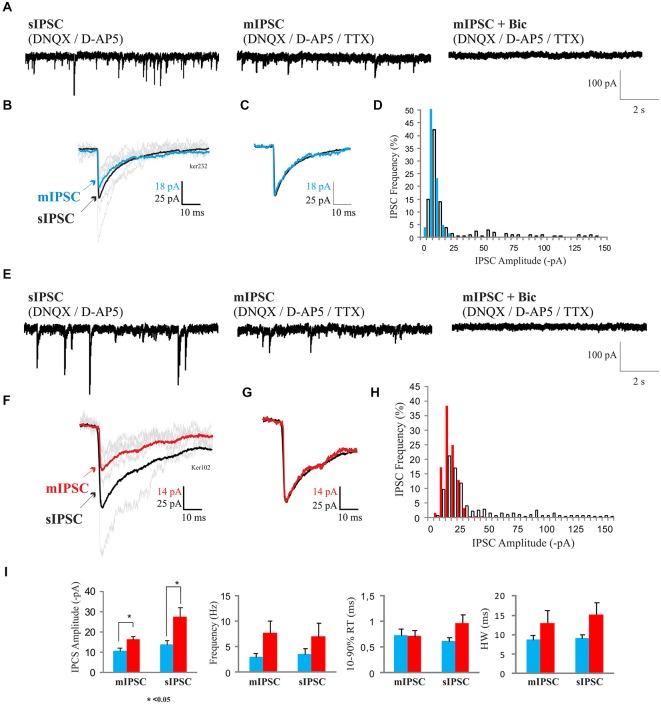
**(A–C,E–G)** IPSCs in developing MSNs. **(A)** Representative recordings of sIPSCs in 7–8 DIV MSNs (*left trace*) in the presence of DNQX/D-AP5, and, following tetrodotoxin (TTX) application (1 μM), of mIPSCs, in the absence (*middle trace*) or presence (*right trace*) of bicuculline (Bic; 10 μM). The sensitivity to Bic (10 μM, *right trace*) of the mIPSCs confirms that these currents were mediated by GABA_A_ receptors. **(B,C)** Superimposed ensemble averages of IPSCs and mIPSCs in **(B)** and scaled averages in **(C)** to compare time course demonstrate that all sIPSCs and mIPSCs in 7–8 DIV MSNs were similar in shape. **(D)** Histogram showing amplitude distributions for mIPSCs (blue) and sIPSCs (white bars). **(E)** Recordings of sIPSCs in 12–14 DIV MSNs (*left trace*) in the presence of DNQX/D-AP5, and, following TTX application (1 μM), of mIPSCs, in the absence (*middle trace*) or presence (*right trace*) of Bic (10 μM). **(F,G)** Superimposed ensemble averages of spontaneous (black) and miniature (red) IPSCs in **(F)** and scaled averages in **(G)** to compare time course demonstrate that all sIPSCs and mIPSCs in 12–17 DIV MSNs were similar in shape. **(H)** IPSC amplitude distributions for mIPSCs (red) and sIPSCs (white bars). For averaged records **(B,C,F,G)** only those events that did not overlap with other incoming events caused by repetitive firing properties of MSNs were selected, while histograms **(D,H)** represent distributions of mIPSC and sIPSC amplitudes of all the events recorded from a representative cell. **(I)** Bar graphs representing amplitudes (far left), frequencies (left), rise times (right) and half-width measurements (far right) of responses. sIPCSs and mIPSCs amplitudes were increased from 7–8 DIV (*n* = 6) to 12–17 DIV (*n* = 5; *p < 0.05, Two sample *t*-test).

sIPSCs were apparent in 20:56 5–7 DIV neurons and 37:43 12–14 DIV neurons recorded. However, in many recordings the frequencies of sIPSCs were too low and recordings were too brief to allow confident analysis of these events. Therefore, recordings selected for the analysis (7–8 DIV, *n* = 6; 12–14 DIV, *n* = 5) were the longer recordings of highest quality. sIPSC frequencies in older cultures (6.3 ± 2.7 s^−1^, mean ± s.e.m., *n* = 5) were similar to sIPSC frequencies in younger cultures (3.5 ± 1.2 s^−1^, mean ± s.e.m.; *n* = 6; Figure 4I). sIPSCs appeared to be briefer at 7–8 DIV (RT 0.6 ± 0.07 ms, HW 9.4 ± 1.1 ms; mean ± s.e.m.) than at 12–14 DIV (RT 0.95 ± 0.17 ms, HW 15.8 ± 3.8 ms; mean ± s.e.m.), but these differences did not reach significance (*p* > 0.05; Figure 4I). However, sIPSC amplitudes were larger in older cultures (7–8 DIV 14.6 ± 2.4 pA, *n* = 6; 12–14 DIV 26.1 ± 3.8 pA, *n* = 5; mean ± s.e.m., *p* < 0.05; Figure 4I). In two young and one older cultures, Bic (10 μM, Figures 4A,E) and in two young and two older cultures, Pic (10 μM, data not shown) blocked spontaneous synaptic activity, confirming that these events were GABA_A_ receptor-mediated. It should be noted that all recordings were made in DNQX which may have depolarized the MSNs by a few mV (Lee et al., 2010), leading to an increase in spontaneous firing and the frequencies of the larger, AP-driven sIPSCs. However, comparisons remain valid since all IPSC recordings were conducted in D-AP5 and DNQX.

sIPSC amplitude distributions in older cultures were often skewed, suggesting that they consisted of a large population of small miniature events, possibly corresponding to AP-independent, single quantum, mIPSCs and a smaller population of larger AP-driven events that might correspond to release from several axon terminals supplied by and simultaneously activated by an AP in a single axon (Figure 4H). To test this, TTX, 1 μM was added to the bath to block APs. In 7–8 DIV cultures, the mIPSC amplitudes were significantly smaller than in older cultures (11.3 ± 1.7 pA, *n* = 6, vs. 17.2 ± 1.5 pA; mean ± s.e.m.; *n* = 5; *p* < 0.05), while the mIPSC frequencies (2.7 ± 0.7, *n* = 6 vs. 7.9 ± 2.7 s^−1^ mean ± s.e.m., *n* = 5; *p* > 0.05) appeared smaller, but these differences did not reach significance (Figure 4I). In older cultures, the mean sIPSC amplitudes (26.1 ± 3.8 pA vs. 17.2 ± 1.5 pA, mean ± s.e.m.; *n* = 5) appeared reduced in TTX (Figure 4F), but these differences did not reach significance (Figures 4F,I). Overall, there appeared to be an increase in the proportion of larger, TTX-sensitive events between 7–8 and 12–14 DIV, without a significant change in the frequency of mIPSCs (Figure 4I). This suggests that as the cultures mature, the synaptic terminals acquire the ability to support AP-driven transmitter release, without a decrease in the frequency of AP-independent release. Spontaneous circuit activity was apparent in some paired recordings in which large, spontaneous bursts of summing IPSCs (or depolarizing IPSPs recorded in current clamp), large enough to elicit APs or break through “action currents”, coincided in both cells. These events were not as tightly synchronized as the coincident sIPSCs seen in some paired recordings.

### Developmental Changes in MSN Synapses

To investigate how the structural properties of synaptic connections between MSNs change over the same time period, we determined the number of presynaptic GABAergic terminals, and the number and size of postsynaptic GABA_A_ receptor clusters per 20 μm length of primary dendrites using immunolabeling and confocal microscopy. In this analysis, we defined and independently analyzed different categories of dendritic postsynaptic GABA_A_ clusters based on the type of the α subunit present in the cluster (α1 or α2). This was based on the results of the *in vivo* study of dendritic synapses received by MSNs in the adult basal ganglia, which has demonstrated that α1- and α2-containing GABA_A_ receptors are the most abundant subtypes present in these synapses (Gross et al., 2011). While imaging these neurons, we have noticed that some α1 and α2 clusters were in a close proximity to each other, thus showing partial overlap in labeling. These clusters were categorized as mixed α1/α2 postsynaptic clusters and analyzed as a separate category. In addition, we have noticed that α1 subunit was also detected as diffuse staining between the clusters along the processes and on the surface of the cell body, particularly at 7 DIV. The number and size of synaptic α1-, α2- or α1/α2-immunolabeled clusters co-localized with presynaptic GAD-65 positive terminals, the overall number of GABA_A_ receptor clusters in each group, and the total number of presynaptic terminals making contacts with MSN dendrites were determined using the Zen 2009 software. Closely associated presynaptic GAD-65 positive terminals and postsynaptic GABA_A_ receptor clusters were considered to define the GABAergic synaptic contacts between MSNs as described in “Material and Methods” Section.

**Figure 5:**
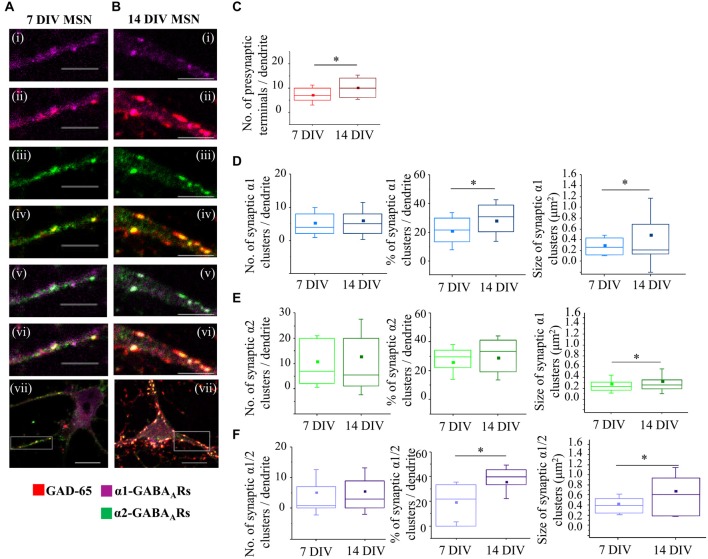
**Developmental changes in GABAergic MSN synapses. (A,B)** Immunolabeling of GABA_A_ receptor α1- (**i,ii,vii**; pink), α2-(**iii,iv,vii**; green), α1/α2- (**v,vi,vii**; white) subunit-containing GABA_A_ receptor clusters, and presynaptic GABAergic terminals (**ii,iv,vi,vii**; red) along the primary dendrites of 7 and 14 DIV MSNs, respectively. (**i–vi**) Scale bar = 5 μm. (**vii**) Scale bar = 10 μm. **(C)** Increase in the number of GABAergic terminals making connections with primary dendrites of MSNs from 7 to 14 DIV (*n* = 23 dendrites at 7 DIV, *n* = 18 dendrites at 14 DIV). **(D–F)** Changes in the number (*left panel*), percentage (*middle panel*) and size (*right panel*) of synaptic α1-, α2-, and α1/α2-clusters, respectively, along the primary dendrites of MSNs from 7 to 14 DIV. The box plots display the median and IQRs of indicated synaptic parameters measured along the first 20 μm of primary dendrites (*n* = 45 dendrites at 7 DIV, *n* = 48 dendrites at 14 DIV) of total of *n* = 16 neurons from two independent experiments. Statistical analysis was performed using Mann Whitney test, *p < 0.05.

Between 7 and 14 DIV, synaptic connections formed between MSNs undergo prominent changes in their number and structural properties (Figures 5A,B). The total number of GAD-65-positive terminals innervating the proximal dendrites (the first 20 μm from the cell body) increased significantly from 7.1 ± 0.9 (mean ± s.e.m., *n* = 23 dendrites) at 7 DIV to 10.3 ± 1.2 (mean ± s.e.m., *n* = 18 dendrites) at 14 DIV (*p* < 0.05, Two sample *t*-test; Figure 5C). In parallel with changes in presynaptic inputs, the number of synaptic GABA_A_ receptor clusters and the percentage of these clusters in the population of all GABA_A_ receptor clusters (synaptic and extrasynaptic) also changed but in a subtype-specific manner.

Thus, the median number of synaptic α1-clusters showed no significant change between 7 and 14 DIV (Figure 5D, left panel—7 DIV: 4 (IQR = 2–8.5; *n* = 45 dendrites), 14 DIV: 5 (IQR = 2–8; *n* = 48 dendrites); *p* value > 0.05, Mann Whitney test), while the median number of all α1-clusters was decreased (7 DIV: 18 (IQR = 10–25; *n* = 45 dendrites), 14 DIV: 11.5 (IQR = 5.25–17.75; *n* = 48 dendrites); *p* value < 0.05, Mann Whitney test, data not shown). Consequently, the percentage of synaptic α1-clusters was increased from 21.4% (IQR = 11.13–30; *n* = 45 dendrites) to 30.8% (IQR = 19.19–38.88; *n* = 48 dendrites), and this increase was statistically significant (*p* value < 0.05, Mann Whitney test; Figure 5D, middle panel). Thus, despite the apparent stability in the number of synaptic α1-clusters, their contribution to the overall number of α1-clusters was significantly increased from 7 to 14 DIV due to down-regulation of extrasynaptic α1-clusters.

The size of synaptic α1-clusters was significantly decreased between 7 and 14 DIV (Figure 5D, right panel). At 7 DIV, the median size of synaptic α1-clusters was 0.3 μm^2^ (IQR = 0.1–0.4; *n* = 194 clusters, *n* = 45 dendrites) compared with 0.2 μm^2^ (IQR = 0.1–0.7; *n* = 250 clusters, *n* = 48 dendrites) at 14 DIV (*p* value < 0.05, Mann Whitney test), while the median size of all α1-clusters showed no significant difference (*p* value > 0.05, Mann Whitney test; data not shown). Together, the data demonstrates that, while the number of dendritic α1-synapses is determined from early stages of MSN differentiation *in vitro*, the size of the postsynaptic GABA_A_ receptors clusters is gradually decreased, suggesting that these synapses undergo functional down-regulation during MSN development.

However, the synapses containing the α2-GABA_A_ receptors showed the opposite trend during differentiation of MSNs *in vitro*. While neither the median number nor the percentage of synaptic α2-clusters showed a significant change (Figure 5E, left and middle panel), the median size of these clusters was significantly increased during differentiation of MSNs *in vitro* (Figure 5E, right panel—7 DIV: 0.2 μm^2^ (IQR = 0.2–0.3; *n* = 492 clusters, *n* = 45 dendrites), 14 DIV: 0.3 μm^2^ (IQR = 0.2–0.4; *n* = 605 clusters, *n* = 48 dendrites; *p* value < 0.001 Mann Whitney test). Similarly, the median size of all α2-clusters was also increased (7 DIV: 0.2 μm^2^ (IQR = 0.2–0.3; *n* = 1216 clusters, *n* = 45 dendrites), 14 DIV: 0.3 μm^2^ (IQR = 0.2–0.4; *n* = 916 clusters, *n* = 48 dendrites; *p* value < 0.001, Mann Whitney test; data not shown). Thus, the number of dendritic α2-synapses is also determined from the early stages of MSN development, but the size of their postsynaptic GABA_A_ receptor clusters is progressively increased, suggesting that these synapses undergo functional up-regulation during MSN development.

Finally, changes in synaptic and total α1/α2-clusters of GABA_A_ receptors along the first 20 μm of primary MSN dendrites were also analyzed (Figure 5F), demonstrating that both the percentage and the size of these clusters were significantly increased between 7 and 14 DIV. Thus, while the median number of synaptic α1/α2-clusters was apparently increased, albeit without reaching statistical significance (Figure 5F, left panel), the median number of all α1/α2-clusters was significantly reduced (7 DIV: 8 (IQR = 4–18; *n* = 45 dendrites), 14 DIV: 6 (IQR = 0–12; *n* = 48 dendrites); *p* value < 0.05, Mann Whitney test, data not shown). Consequently, the percentage of synaptic α1/α2-clusters was increased from 21.7% (IQR = 0–33.33; *n* = 45 dendrites) at 7 DIV to 39.6% (IQR = 33.3–45.45; *n* = 48 dendrites) at 14 DIV (*p* value < 0.001; Mann Whitney test, Figure 5F, middle panel), probably due to down-regulation of the extrasynaptic α1/α2-clusters.

These synaptic α1/α2-clusters were significantly larger than the individual α1 or α2 clusters and they underwent a significant increase in size as MSNs matured, with the median size of 0.4 μm^2^ (IQR = 0.2–0.5; *n* = 234 clusters, *n* = 45 dendrites) at 7 DIV compared with 0.6 μm^2^ (IQR = 0.9–0.2; *n* = 234 clusters, *n* = 48 dendrites) at 14 DIV (*p* value < 0.001, Mann Whitney test; Figure 5F, right panel). The increase in size was also observed across the whole population of α1/α2-clusters (7 DIV: the median size of 0.4 μm^2^ (IQR = 0.3–0.6; *n* = 560 clusters, *n* = 45 dendrites, 14 DIV: median size of 0.6 μm^2^ (IQR = 0.2–0.9; *n* = 384 clusters; *n* = 48 dendrites); *p* value < 0.001, Mann Whitney test; data not shown). The data demonstrates a prominent up-regulation of mixed α1/α2 synaptic clusters during MSN development in culture.

We have thus revealed that in developing MSNs dendritic α1-, α2- and α1/α2**-**GABA_A_ receptor clusters undergo specific changes in their number and size in parallel with a prominent increase in the number of GABAergic nerve terminals innervating MSN dendrites.

### Formation of Gephyrin Clusters in Developing MSNs

**Figure 6:**
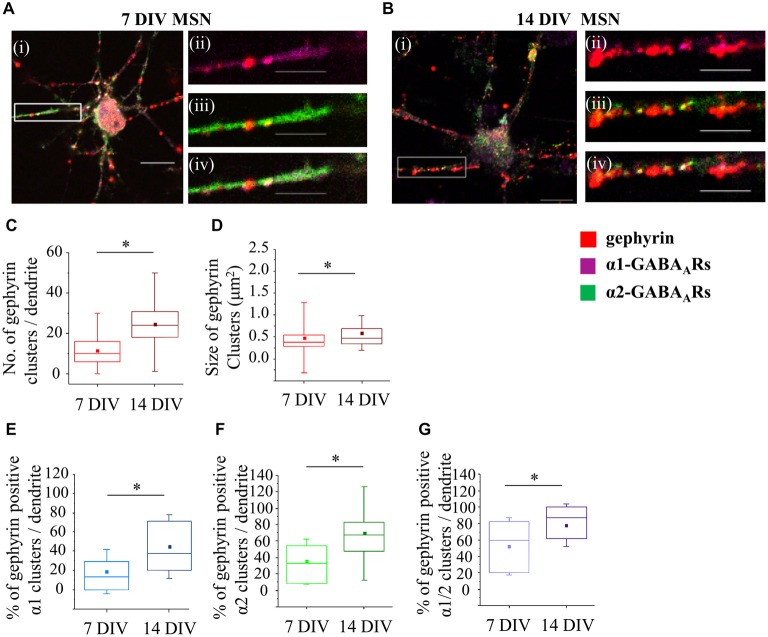
**Developmental changes in gephyrin clustering at GABAergic synapses of MSNs. (A,B)** Immunolabeling of gephyrin clusters (**i–iv**; red) and α1- (**ii**; pink), α2- (**iii**; green) and α1/α2- (**iv**; white) subunit-containing clusters along the primary dendrites of 7 and 14 DIV MSNs, respectively. **(i)** Scale bar = 10 μm. **(ii–iv)** Scale bar = 5 μm. **(C,D)** Increase in the number and size, respectively, of gephyrin clusters along the primary dendrites of MSNs from 7 to 14 DIV. **(E,F,G)** Increase in the percentage of gephyrin clusters colocalized with GABA_A_R α1-, α2- and α1/α2- clusters, respectively, along the primary dendrites of MSNs from 7 to 14 DIV. The box plots display the median and IQRs of indicated synaptic parameters measured along the first 20 μm of primary dendrites (*n* = 78 dendrites at 7 DIV, *n* = 70 dendrites at 14 DIV) of total of *n* = 16 neurons from two independent experiments. Statistical analysis was performed using Mann Whitney test, *p < 0.05.

It is now well established that the scaffolding protein gephyrin plays a central role in the maintenance and stability of GABA_A_ receptor clusters (Tyagarajan and Fritschy, 2014). To characterize changes in gephyrin association with GABA_A_ receptors α1-, α2- or α1/α2-clusters during development, we have performed immunolabeling with specific antibodies and confocal imaging (Figures 6A,B). Initially, we have estimated the number and the size of gephyrin clusters along the first 20 μm of primary dendrites. At 7 DIV, the median number of gephyrin clusters was significantly increased from 10 (IQR = 6–16; *n* = 78 dendrites) to 24 (IQR = 18–3; *n* = 70 dendrites) at 14 DIV (*p* value < 0.001, Mann Whitney test; Figure 6C). The size of gephyrin clusters was also significantly increased from 0.4 μm^2^ (IQR = 0.3–0.5; *n* = 914 clusters, *n* = 78 dendrites) at 7 DIV to 0.5 μm^2^ (IQR = 0.3–0.7; *n* = 1706 clusters, *n* = 70 dendrites) at 14 DIV (*p* value < 0.001, Mann Whitney test; Figure 6D). These changes were accompanied with a significant increase in the percentage of α1-, α2- or α1/α2-clusters associated with gephyrin during development. Thus, at 7 DIV, 13.7% (IQR = 0–30.6; *n* = 72 dendrites) of all α1-clusters was associated with gephyrin, compared with 37.5% (IQR = 20–72; *n* = 65 dendrites) at 14 DIV (*p* value < 0.00001, Mann Whitney test; Figure 6E). A substantially larger number of α2-clusters associated with gephyrin at 7 DIV, i.e., 33.3% (IQR = 8.2–54.8; *n* = 74 dendrites) of all α2-clusters, was increased to 67.5% (IQR = 46.6–83.3; *n* = 68 dendrites) at 14 DIV (*p* value < 0.000001, Mann Whitney test; Figure 6F). Finally, the percentage of α1/α2-clusters associated with gephyrin was also significantly increased (7 DIV: 60% (IQR = 20–81.8; *n* = 71 dendrites), 14 DIV: 87.5% (IQR = 61.1–100; *n* = 66 dendrites), *p* value < 0.001, Mann Whitney test; Figure 6G). Together, the data indicates that gephyrin association with GABAergic synapses is gradually increased during MSN development. However, this does not correlate tightly with changes observed in either the number or the size of postsynaptic GABA_A_ receptor clusters, as α1-clusters underwent a significant reduction in size despite the fact that their association with gephyrin was increased from 7 to 14 DIV.

### Developmental Switch in GABA_A_ Receptor Signaling in MSNs

**Figure 7:**
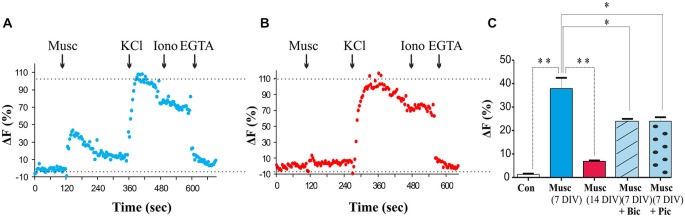
**Developmental switch in GABA_A_ receptor signaling in MSNs. (A,B)** Changes in intracellular Ca^2+^ in response to muscimol (50 μM), KCl (60 mM), ionomycin (5 μM) and EGTA (1 mM) in 5–7 DIV and 12–14 DIV MSNs, respectively, measured by fluorescent microscopy. **(C)** The histogram shows the maximum increase in ΔF for control and muscimol alone or in the presence of bicuculline (+ Bic) or picrotoxin (+ Pic), normalized to the peak response to KCl application. Bars represent mean ± s.e.m. Statistical analysis was performed using ANOVA, *p < 0.01, **p < 0.001.

The role of GABA as a developmental signal has been well established in many brain regions, where activation of GABA_A_ receptors triggers depolarization and influx of Ca^2+^ (Cancedda et al., 2007; Porcher et al., 2011; Ben-Ari et al., 2012; Deidda et al., 2015). To assess whether GABA_A_ receptor activation leads to plasma membrane depolarization and Ca^2+^ influx in MSNs at 7 DIV, a membrane-permeable Ca^2+^ indicator fluo-4-AM was applied to the cultures. Ca^2+^ levels were monitored in response to muscimol (50 μM), a specific GABA_A_ receptor agonist, using fluorescence microscopy as described in “Materials and Methods” Section. A muscimol-evoked increase in [Ca^2+^]_i_ defined as fluorescence intensity at least 50% above the baseline fluorescence, was observed in 50% of cells tested (23 of 46 cells tested, *P* < 0.05, paired *t*-test vs. vehicle; *n* = 23; Figure 7A). All cells examined exhibited [Ca^2+^]^i^ responses to depolarization evoked by high extracellular K^+^(60 mM) with no significant difference in the maximal change in fluorescence between those cells insensitive or sensitive to muscimol (*P* > 0.05, Paired *t*-test; *n* = 23). Total Ca^2+^ levels were assessed by the addition of ionomycin (5 μM) followed by the addition of the Ca^2+^ chelator EGTA (Figure 7A). Muscimol-induced Ca^2+^ responses were significantly attenuated in the presence of the GABA_A_ receptor competitive antagonist Bic or a channel blocker Pic (both at 50 μM; *P* < 0.01, paired *t*-test; *n* = 20 (Figure 7C), but not abolished, as it is possible that some GABA_A_ receptors were not completely blocked at these concentrations of antagonists. Bic had no significant effect on basal fluorescence when added alone (*P* > 0.05, paired *t*-test, *n* = 20; data not shown).

During the period of MSN maturation (7–14 DIV), a switch in the functional outcome of GABA_A_ receptor activation from depolarization to hyperpolarization was observed resulting in a loss of muscimol-dependent increase in intracellular Ca^2+^ (Figure 7B). These data suggest that activation of GABA_A_ receptors in immature MSNs leads to depolarization and voltage-gated Ca^2+^ influx likely through the voltage-gated calcium channel (VGCC; Porcher et al., 2011). However, as the neurons mature, depolarizing activity of GABA_A_ receptors declines and is superseded by hyperpolarization of the plasma membrane by these receptors at 14 DIV.

### GABA_A_ Receptor Activity has no Influence on MSN Synapses Early in Development

**Figure 8:**
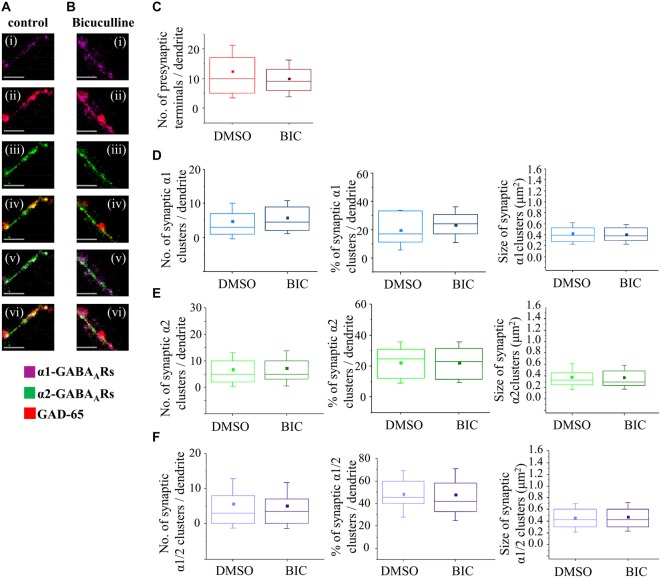
**GABA_A_ receptor activity has no influence on synapse formation between immature MSNs. (A,B)** Immunolabeling of GABA_A_ receptor α1- (**i,ii**; pink), α2- (**iii,iv**; green), α1/α2- (**v,vi**; white) subunit-containing clusters, and presynaptic GABAergic terminals (**ii, iv, vi**; red) along the primary dendrites of 7 DIV MSNs in the presence of vehicle control dimethy sulfoxide (DMSO) or Bic (25 μM), respectively. **(i–vi)** Scale bar = 5 μm. **(C)** The number of GABAergic terminals forming connections with primary dendrites of MSNs (*n* = 43 dendrites in DMSO/controls, *n* = 50 dendrites in Bic-treated cultures). **(D–F)** The number, percentage and size of synaptic α1-, α2- and α1/α2-clusters, respectively, along the primary dendrites of MSNs. The box plots display the median and IQRs of indicated synaptic parameters measured along the first 20 μm of primary dendrites (*n* = 49 dendrites in DMSO/controls, *n* = 46 dendrites in Bic-treated cultures) of total of *n* = 16 neurons from two independent experiments. Statistical analysis was performed using Mann Whitney test, *p < 0.05.

To establish whether depolarising activity of GABA_A_ receptors has a regulatory role in synapse formation at the early stage of MSN development, and contrast this with the hyperpolarizing activity of GABA_A_ receptors at the later stage, we have applied a chronic blockade of GABA_A_ receptors using a competitive antagonist Bic (25 μM) in MSN cultures from 4–7 DIV or 7–14 DIV. We have first established that, at this concentration of Bic, the survival of MSNs was not impaired (data not shown). Using the immunocytochemistry approach and analysis described above, we subsequently characterized the formation of GABAergic synapses and the number and size of postsynaptic GABA_A_ receptor clusters (Figures 8A,B).

The median number of GAD-65-positive terminals making contacts with the primary dendrites of MSNs (the first 20 μm) was estimated following the treatment with the vehicle (10, IQR = 5–17, *n* = 43 dendrites; DMSO) or Bic (9, IQR = 6–13; *n* = 50 dendrites) from 4 to 7 DIV (Figure 8C). No significant change was detected following these treatments (*p* value > 0.05, Mann Whitney test) indicating that connectivity between MSNs is not influenced by GABA_A_ receptors activity early in development.

To investigate whether GABA_A_ receptor activity plays a role in proper positioning and the assembly of different types of inhibitory synapses, we have estimated the number and the size of postsynaptically localized GABA_A_ receptor clusters containing α1, α2 or α1/α2 subunits, and their contribution to the overall population of GABA_A_ receptor clusters following the treatments described above.

The median number of synaptic α1-clusters showed an apparent up-regulation although this was not statistically significant (DMSO control: 3 (IQR = 1–7; *n* = 49 dendrites), Bic: 4.5 (IQR = 2–9.2; *n* = 46 dendrites), *p* value > 0.05, Mann Whitney test; Figure 8D, left panel). Similarly, the median number of all α1-clusters (data not shown) and the percentage of synaptic α1-clusters in this population did not change significantly following these treatments (DMSO/control: 16.7% (IQR = 10.1–33.3; *n* = 49 dendrites), Bic: 24.1% (IQR = 16.0–30.9; *n* = 46 dendrites); *p* value > 0.05, Mann Whitney test; Figure 8D, middle panel).

The median size of synaptic α1-clusters did not change in response to chronic blockade of GABA_A_ receptors (Figure 8D, right panel—DMSO/control: 0.4 μm^2^ (IQR = 0.3–0.5; *n* = 194 clusters, *n* = 49 dendrites), Bic: 0.4 μm^2^ (IQR = 0.3–0.5; *n* = 231 clusters, *n* = 46 dendrites); *p* value > 0.05, Mann Whitney test), and, similar results were obtained when all α1-clusters were analyzed (*p* value > 0.05, Mann Whitney test, data not shown).

We were also interested to analyze the number and the size of dendritic α2-clusters following the treatment of neurons with vehicle or Bic from 4–7 DIV (Figure 8E). The median number of synaptic α2-clusters was 5 (IQR = 2–11; *n* = 49 dendrites) in vehicle-treated neurons compared with 5 (IQR = 3–10; *n* = 46 dendrites) in Bic-treated neurons (*p* > 0.05, Mann Whitney test; Figure 8E, left panel). Similarly, no change was observed in the median number of all α2-clusters (*p* value > 0.05, Mann Whitney test; data not shown) and, consequently, in the percentage of synaptic α2-clusters in the population of all α2-clusters (Figure 8E, middle panel; *p* > 0.05, Mann Whitney test).

The median size of synaptic α2-clusters was 0.3 μm^2^ (IQR = 0.2–0.4; *n* = 328 clusters, *n* = 49 dendrites) in controls compared with 0.3 μm^2^ (IQR = 0.2–0.5; *n* = 366 clusters, *n* = 46 dendrites) in Bic-treated cells (*p* value > 0.05; Mann Whitney test), indicating no significant change (Figure 8E, right panel). However, a significant decrease in the median size of all α2-clusters was observed (DMSO/control: 0.3 μm^2^ (IQR = 0.2–0.4; *n* = 745 clusters, *n* = 49 dendrites, Bic: 0.2 μm^2^ (IQR = 0.2–0.4; *n* = 741 clusters, *n* = 46 dendrites; *p* value < 0.001, Mann Whitney test, data not shown), suggesting that this change may be due to a decrease in the size of the extrasynaptic α2-clusters following GABA_A_ receptor blockade at this developmental stage.

Finally, the number and size of synaptic α1/α2-clusters were estimated. No significant change in the number of synaptic α1/α2-clusters (Figure 8F, left panel), all α1/α2-clusters (data not shown) and, consequently, the percentage of synaptic α1/α2-clusters (Figure 8F, middle panel), was observed under the chronic blockade of GABA_A_ receptor activity with Bic (*n* = 49 dendrites in controls, *n* = 46 dendrites in Bic-treated samples).

The median size of synaptic α1/α2-clusters was 0.4 μm^2^ (IQR = 0.3–0.6; *n* = 271 clusters, *n* = 49 dendrites) in controls compared with 0.4 μm^2^ (IQR = 0.3–0.6; *n* = 242 clusters, *n* = 46 dendrites) in Bic-treated cells (*p* value > 0.05, Mann Whitney test; Figure 8F, right panel). Similarly, the median size of all α1/α2-clusters was 0.4 μm^2^ (IQR = 0.3–0.6; *n* = 456 clusters, *n* = 49 dendrites) in controls vs. 0.4 μm^2^ (IQR = 0.3–0.6; *n* = 447 clusters, *n* = 46 dendrites) in Bic-treated cells (*p* value > 0.05, Mann Whitney test; data not shown). Together, the data demonstrates that the number of presynaptic GAD-65 positive terminals, and the size and the number of synaptically localized GABA_A_ receptor clusters containing α1, α2 or α1/α2 subunits, are not influenced by the activity of GABA_A_ receptors early in development when this activity leads to depolarization and increased intracellular Ca^2+^ concentration.

### GABA_A_ Receptor Activity Regulates the Formation of GABAergic Synapses Later in Development

**Figure 9:**
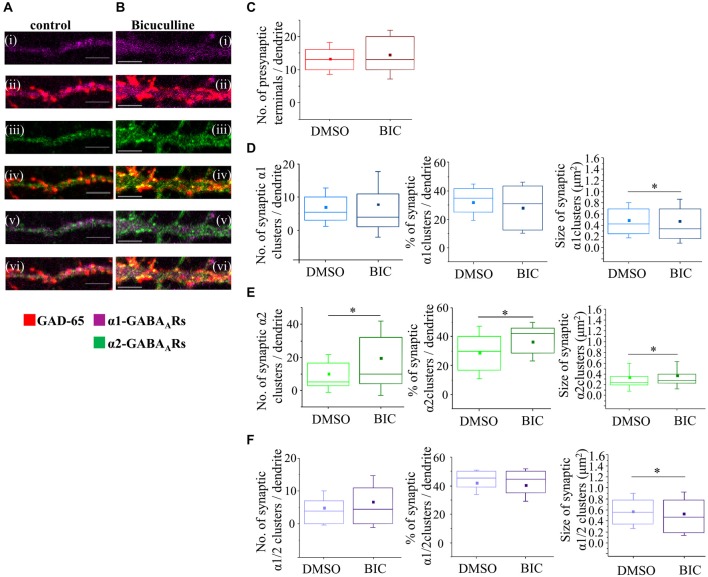
**GABA_A_ receptor activity regulates synapse formation between mature MSNs. (A, B)** Immunolabeling of GABA_A_ receptors α1- (**i,ii**; pink), α2- (**iii,iv**; green), α1/α2- (**v,vi**; white) subunit-containing clusters, and presynaptic GABAergic terminals (**ii,iv,vi**; red) along the primary dendrites of 14 DIV MSNs in the presence of vehicle control dimethy sulfoxide (DMSO) or Bic (25 μM), respectively. **(i–vi)** Scale bar = 5 μm. **(C)** The number of GABAergic terminals forming connections with primary dendrites of MSNs (*n* = 74 dendrites in DMSO/controls, *n* = 82 dendrites in Bic-treated cultures). **(D–F)** The number, percentage and size of synaptic α1-, α2- and α1/α2-clusters, respectively, along the primary dendrites of MSNs. The box plots display the median and IQRs of indicated synaptic parameters measured along the first 20 μm of primary dendrites (*n* = 84 dendrites in DMSO/controls, *n* = 80 dendrites in α1 and α1/α2-cluster analysis and *n* = 79 in α2 cluster analysis) of total of *n* = 16 neurons from two independent experiments. Statistical analysis was performed using Mann Whitney test, *p < 0.05.

To characterize the role of GABA_A_ receptor activity in synapse formation at later stages of MSN development when these receptors mediate hyperpolarization of the plasma membrane (Figure 7B), we treated cultured MSNs with vehicle (DMSO) or Bic (25 μM) from 7 to 14 DIV, and examined the properties of their synaptic contacts using immunolabeling and confocal microscopy as described above (Figures 9A,B).

The total number of GAD-65-positive terminals forming contacts with the primary dendrites was first estimated (Figure 9C). In control, vehicle-treated cultures, the median number of presynaptic terminals was 13 (IQR = 10–20; *n* = 74 dendrites) compared with 13 (IQR = 9–16; *n* = 82 dendrites) in Bic-treated cultures (*p* value > 0.05, Mann Whitney test; Figure 9C).

The median number of synaptic α1-clusters was 5.5 (IQR = 3–10; *n* = 84 dendrites) in controls compared to 4 (IQR = 1–11; *n* = 80 dendrites) in Bic-treated neurons, but this change did not reach significance (*p* value > 0.05, Mann Whitney test; Figure 9D, left panel). The median number of all α1-clusters (data not shown) and the percentage of synaptic α1-clusters in this population (Figure 9D, middle panel) also remained unaltered under the chronic blockade of GABA_A_ receptors with Bic.

However, the median size of synaptic α1-clusters was significantly decreased from 0.4 μm^2^ (IQR = 0.2–0.7; *n* = 481 clusters, *n* = 84 dendrites) in controls to 0.3 μm^2^ (IQR = 0.2–0.7; *n* = 576 clusters, *n* = 80 dendrites; *p* value < 0.05 Mann Whitney test; Figure 9D, right panel) in Bic-treated neurons. Similarly, the median size of all α1-clusters was also significantly decreased from 0.4 μm^2^ (IQR = 0.2–0.6; *n* = 940 clusters, *n* = 84 dendrites) in controls to 0.3 μm^2^ (IQR = 0.2–0.7; *n* = 898 clusters, 80 dendrites) in Bic-treated neurons (*p* value < 0.01, Mann Whitney test; data not shown).

In contrast to changes in synaptic α1-clusters, the median number, percentage and size of synaptic α2-clusters were significantly increased in response to chronic inhibition of GABA_A_ receptors with Bic (Figure 9E). Thus, the median number of these clusters was significantly increased from 5 (IQR = 3–17; *n* = 84 dendrites) in vehicle-treated neurons to 10 (IQR = 4–32; *n* = 79 dendrites) in Bic-treated neurons (*p* value < 0.05, Mann Whitney test; Figure 9E, left panel), while the median number of all α2-clusters was 16 (IQR = 9–31; *n* = 84 dendrites) in controls compared with 18 (IQR = 9–40; *n* = 79 dendrites) in Bic-treated neurons (*p* value > 0.05, Mann Whitney test; data not shown). The estimated percentage of synaptic α2-clusters among all α2-clusters was therefore significantly increased from 30% (IQR = 16–40; *n* = 84 dendrites) in controls to 42% (IQR = 29–43; *n* = 79 dendrites) in the Bic-treated neurons (*p* value < 0.001, Mann Whitney test; Figure 9E, middle panel).

The median size of synaptic α2-clusters was also significantly increased following the treatment with Bic (Figure 9E, right panel—DMSO/control: 0.2 μm^2^ (IQR = 0.2–0.3; *n* = 859 clusters, *n* = 84 dendrites), Bic: 0.3 μm^2^ (IQR = 0.2–0.4; *n* = 1546 clusters, *n* = 79 dendrites), *p* value < 0.001, Mann Whitney test). Similarly, the median size of all α2 clusters was significantly increased from 0.2 μm^2^ (IQR = 0.2–0.3; *n* = 1397 clusters, *n* = 84 dendrites) in controls to 0.3 μm^2^ (IQR = 0.2–0.4; *n* = 2038 clusters, *n* = 79 dendrites) in Bic-treated neurons (*p* value < 0.001; Mann Whitney test; data not shown).

Finally, the median number of synaptic or all α1/α2-clusters, and the percentage of synaptic α1/α2-clusters, remained unaltered under the chronic blockade of GABA_A_ receptors with Bic (Figure 9F, left and middle panel; *n* = 84 dendrites in DMSO/controls, *n* = 80 dendrites in Bic-treated samples). However, the median size of synaptic α1/α2-clusters was significantly decreased from 0.6 μm^2^ (IQR = 0.3–0.8; *n* = 409 clusters, *n* = 84 dendrites) in controls to 0.5 μm^2^ (IQR = 0.2–0.8; *n* = 518 clusters, *n* = 80 dendrites) in Bic-treated neurons (*p* value < 0.05, Mann Whitney test; Figure 9F, right panel). Similarly, the median size of all α1/α2-clusters was decreased from 0.6 μm^2^ (IQR = 0.3–0.8; *n* = 539 clusters, *n* = 84 dendrites) in controls compared with 0.5 μm^2^ (IQR = 0.2–0.7; *n* = 729 clusters, *n* = 80 dendrites) in Bic-treated neurons (*p* value < 0.001; Mann Whitney test; data not shown).

Thus, the formation and maturation of two main subtypes of GABAergic synapses formed by MSNs are regulated by GABA_A_ receptor activity, but in opposite directions, and thus appear to be driven by different mechanisms.

## Discussion

The vast majority of neurons that form the basal ganglia are GABAergic medium spiny neurons, the main projection neurons that form direct output pathways to the brain stem to control motor function, and to the thalamus and cortex to regulate behavior, emotions and cognition. In this study, we have investigated how the functional properties and synaptic activity of embryonic precursors of these neurons develop over time *in vitro* and how the chronic inhibition of this activity influences the type and the structure of synaptic connections formed by these neurons. Given that inhibitory synapses between MSNs have a direct effect on the activity and the overall functional output from the basal ganglia, our data provide important insight into the mechanisms that regulate the embryonic development of the basal ganglia.

### Functional Maturation and Structural Changes in MSN Synapses

Between 5–7 and 12–14 DIV, the electrophysiological properties of the MSNs matured. APs could be elicited in a minority of younger neurons, but these events were broad and of small amplitude, emerged from the rising phase of the voltage response to current injection, with no interruption in the trajectory and the neurons did not support repetitive firing. Whether these low amplitude APs are simply the result of low Na^+^ channel density, or a distant spike-initiation zone and passive reflection of an axonal spike at the soma, is unclear. However, this behavior may correlate with the low incidence of larger, putatively AP-driven, sIPSCs, in these young cultures. By 14 DIV, all neurons supported APs, though these were still of smaller amplitude that is typical of fully mature neurons (Kawaguchi et al., 1989; Nisenbaum et al., 1994; Lepora et al., 2011; Pidoux et al., 2011). Thus, embryonic MSNs steadily acquired characteristics typical of adult MSNs reported previously, though they did not quite match them by 14 DIV. The slow depolarizing ramp typical of *ex vivo* MSNs and due, at least in part, to a slowly inactivating K^+^-current (Nisenbaum et al., 1994; Falk et al., 2006), appeared as neurons acquired the ability to maintain repetitive firing (Figure 3). Membrane time constants increased, possibly in parallel with an increase in size and dendritic tree complexity (Lee and Sawatari, 2011), and spike AHPs increased in amplitude. Inward rectification in responses to hyperpolarizing pulses was apparent in some neurons at all ages, but was less pronounced than that reported for fully mature *ex vivo* MSNs. The differences in functional properties of MSNs observed in dissociated primary culture can be attributed to the conditions in which neurons are grown *in vitro*, with empirically determined concentrations of growth factors and in the absence of endogenous active molecules. Nevertheless, functional properties and synaptic development of MSNs *in vitro* were similar to those described in more intact preparations as shown previously (Weiss et al., 1986; Kowalski et al., 1995; Falk et al., 2006; Lalchandani and Vicini, 2013), suggesting that these processes are largely driven by predetermined genetic programs (Yun et al., 2003).

Cultured MSNs established functional synapses by 7 DIV. However, the larger spontaneous events were not apparent in younger cultures. Moreover, no demonstrably AP-driven IPSCs were recorded in paired recordings at 5–7 DIV. The frequencies but not the amplitudes of mIPSCs, recorded in TTX were, however, similar to those recorded in older cultures (Figure 4). This suggests that the ability to support AP-driven IPSCs develops later than the ability to release single quanta spontaneously. Although it was assumed, traditionally, that the synaptic vesicles that are responsible for the spontaneous release of single quanta belong to the same pool as those that mediate synchronous, or AP-driven release, there is now overwhelming evidence that two different pools of vesicles, whose release is controlled by quite different mechanisms, are involved (Hablitz et al., 2009; Chung et al., 2010). A third independent pool responsible for asynchronous release has also been proposed (Smith et al., 2012). The facility for AP-driven release does not appear to be dependent upon the time since synapse formation, but rather on the maturity of the presynaptic neuron. Synapse-like contacts between MSNs (E17 cultures, 15 DIV) and GABA_A_ receptor-expressing human embryonic kidney 293 (HEK293) cells, acquire this ability within 24 h (Fuchs et al., 2013).

The simplest explanation for the differences between release modes seen at the different ages here, would be that the pool responsible for mIPSCs can become fully developed by 7 DIV, but that a second pool and/or the mechanisms that are responsible for AP-driven release of that pool, develop later. One function proposed for spontaneous quantal events, is the stabilization of synapses, even in the absence of synchonous release (Smith et al., 2012). This function may be more important during early synapse development than is synchronous release. Whether these synapses possess the mechanisms necessary for asynchronous release, remains unclear. Responses, to what appeared to be single APs, in some paired recordings in which the presynaptic MSN fired fast APs on a depolarizing envelope, included later events that might be considered to be asynchronous releases. However, these events occurred at constant latency following the first AP, suggesting that they were triggered by second, or third APs that were not apparent as distinct events at the soma. There is a possibility that in these still immature neurons, the ability to support high frequency repetitive firing matures in the axon before it matures in the soma.

The stability of mIPSC frequency over time suggests that these younger MSNs may receive as many functional inputs as the older cells despite the observed overall increase in the number of terminals forming contacts with their dendrites from 7 to 14 DIV, implying that a fraction of these terminals is not fully functional or fails to appropriately “wire” into the functional synaptic contacts. However, it is possible that the numbers of GABA_A_ receptors activated by each quantal release are increased during development *in vitro* given that the amplitudes of mIPSCs show a significant increase. Further electrophysiological experiments with a larger number of cells would help establish the real changes in functional properties of MSN synapses. Our preliminary data is, however, in agreement with the switch in properties and composition of GABAergic clusters, with α2-clusters undergoing an increase in size while α1-clusters are concomitantly reduced in the same neuron. It is important to note that as MSNs mature in culture, they acquire not only the functional but also the structural properties of adult MSNs, with the α2-GABAergic clusters being significantly more abundant than α1-clusters in the striatum (Fritschy and Mohler, 1995). This up-regulation of α2-GABAergic clusters occurs in parallel with an increase in their association with the postsynaptic scaffolding protein gephyrin, although the causal relationship between these two processes remains questionable, given that α1-GABAergic clusters also undergo increased association with gephyrin, yet their size is actually decreased. The observed increase in the number and size of gephyrin clusters in MSNs resembles changes reported in other types of neurons, e.g., developing hippocampal pyramidal neurons (Tyagarajan and Fritschy, 2014), and is likely to contribute to the overall stabilization rather than formation of these synapses.

The α1- and α2-containing synapses are likely to show profound functional differences given that the corresponding GABA_A_ receptors have distinct kinetic properties. The α1-GABA_A_ receptors are known to have the fast deactivation and desensitization kinetics (Freund and Buzsáki, 1996; Klausberger et al., 2002) while the α2-GABA_A_ receptors are characterized by rapid activation, slow deactivation rates (Lavoie et al., 1997) and a significantly higher affinity for GABA (Levitan et al., 1988). Thus, the up-regulation of α2 synapses is likely to lead to enhanced collateral inhibition between MSNs and may be finely tuned with the reported silencing of the striatal MSN activity occurring during the second postnatal week (Dehorter et al., 2012). However, a prominent increase in the abundance and size of mixed α1/α2-synapses was also observed. Functional implications of these changes are difficult to predict at present given that the relative abundance of these receptor subunits in individual synapses may vary as previously shown in the analysis of mixed α1/α2 perisomatic synapses of hippocampal pyramidal neurons (Kasugai et al., 2010).

### Depolarization-to-Hyperpolarization Shift in GABA_A_ Receptor Activity and Synaptic Development

In young hippocampal and cortical neurons up to 7 DIV, GABA_A_ receptors exert a depolarizing action due to high intracellular Cl^−^ concentrations. This occurs due to the low expression of the neuronal Cl^−^ extruding K^+^/Cl^−^ co-transporter KCC2 (Rivera et al., 1999). At the time when GABA_A_ receptors are depolarizing, their activation can increase [Ca^2+^]_i_ through the opening of voltage-gated Ca^2+^ channels (Yuste and Katz, 1991; Lin et al., 1994; Owens et al., 1996). During development GABA_A_ receptors become inhibitory, likely due to up-regulation of KCC2, which extrudes Cl^−^ resulting in a lower intracellular Cl^−^ concentration (Ludwig et al., 2003). Similar shift in muscimol-evoked GABA_A_ receptor activation after 7 DIV was observed in the present study, demonstrating that, in MSNs at 7 DIV, activation of GABA_A_ receptors causes a transient rise in [Ca^2+^]^i^ and that this response was subsequently absent in MSNs at 14 DIV (Figure 7). However, these changes were not recorded in a small population of neurons examined at 7 DIV, which may be due to transition of these neurons towards higher expression of KCC2 and consequently lower intracellular Cl^−^ concentration already at this stage (Ludwig et al., 2003). These responses resemble clustered voltage-gated calcium activity reported in early postnatal MSNs which was sensitive to GABA_A_ receptor antagonists and related to the appearance of giant depolarizing potentials (GDPs; Dehorter et al., 2012). The shifts in Ca^2+^ responses observed in culture and in acute slices prepared from animals at equivalent stages of embryonic and early postnatal development (Dehorter et al., 2012), are also well correlated in time, as more mature functional properties of MSNs were observed around 14 DIV in culture which is roughly equivalent to the beginning of the second postnatal week (~P8). The arrival of GABA_A_ receptor-mediated hyperpolarization occurs in concert with morphological (Kowalski et al., 1995; Lee and Sawatari, 2011) and functional changes (Kowalski et al., 1995; Dehorter et al., 2011) in the properties of MSNs, and the ability to coordinate the movement in rats (Dehorter et al., 2011), indicating that the shift is an important step towards functional maturation of MSN circuits that mediate behavior. While the specific types of GABAergic synapses that MSNs form with their targets are the core elements of these circuits, whether and how the activity of GABA_A_ receptors influences their formation and structural characteristics is unclear. In the experiments described here, the chronic inhibition of GABA_A_ receptors in young MSNs, at the time when these receptors cause depolarization, had no detectable effects on the structural properties of α1-, α2- or mixed α1/α2 synapses formed by MSNs. However, blockade of GABA_A_ receptor activity at the stage of transition towards hyperpolarization caused significant reduction in the size of the α1- and mixed α1/α2-synaptic clusters and concomitant increase in the number and size of α2-synaptic clusters indicating a prominent up-regulation of α2-synapses. As the similar up-regulation of α2-synapses was observed during normal development of MSNs in culture and *in vivo*, it appears that GABA acting through GABA_A_ receptors can modulate a predetermined genetic program of synaptic development of MSNs and, therefore, may contribute significantly to the fine tuning of inhibitory connections in the circuitry of the basal ganglia.

## Conflict of Interest Statement

The authors declare that the research was conducted in the absence of any commercial or financial relationships that could be construed as a potential conflict of interest.
